# Prevalence and antimicrobial resistance patterns of urinary tract infections at the university hospital center in Marrakech Morocco (2020–2024): a retrospective study

**DOI:** 10.1186/s12866-026-05425-6

**Published:** 2026-07-24

**Authors:** Zakaria Ellehah, Lahcen Ouahmane, Asmae Lamrani Hanchi, Nabila Soraa

**Affiliations:** 1 Faculty of Sciences-Semlalia, Laboratory of Water Sciences, Microbial Biotechnologies, and Natural Resources Sustainability (AQUABIOTECH), Unit of Microbial Biotechnologies, Agrosciences, and Environment (BIOMAGE) CNRST Labeled Research Unit N°4, Cadi Ayyad University, PO Box 2390, Marrakech, 40000 Morocco; 2 Department of Microbiology, Mohamed VI University Hospital Center, Marrakech, Morocco

**Keywords:** Urinary tract infections, Antimicrobial resistance, Antibiotics, Morocco

## Abstract

**Objective:**

To determine the prevalence, epidemiological and microbiological characteristics of urinary tract infections (UTIs), and to assess antimicrobial resistance (AMR) trends at the University Hospital Center Mohammed VI, Marrakech, over a five-year period.

**Methods:**

A retrospective cross-sectional study was conducted from January 2020 to December 2024. All urine specimens processed at the microbiology laboratory were reviewed. Duplicate isolates were excluded, and only the first positive isolate per patient per infectious episode was retained. Bacterial identification was performed using MALDI-TOF MS, and antimicrobial susceptibility testing was carried out using disk diffusion and the BD Phoenix^®^ automated system. For consistency, AST results were rechecked and interpreted according to a single harmonized CA-SFM/EUCAST 2025 guideline version. Temporal trends in resistance were assessed using beta-regression models, with annual changes expressed as percentage-point variation per year and *p*-values adjusted using the Benjamini-Hochberg false discovery rate procedure.

**Results:**

Of 62,788 urine specimens, 8,231 were culture-positive 13.1%. Prevalence rose from 11.5% in 2020 to 14.9% in 2022, then stabilized at 12–13% in 2023–2024. Women accounted for 59.6% of cases, and patients ≥ 60 years were the most affected. *Escherichia coli* was the leading uropathogens 47.9%, followed by *Klebsiella pneumoniae* 16.7% and *Enterococcus* spp. 4.6%. Significant increases in resistance were observed for several clinically important antimicrobials. Amoxicillin-clavulanate resistance increased significantly in both *E. coli* (+ 6.00% points/year; 95% CI: 2.83–8.82; FDR-*p* < 0.001) and *K. pneumoniae* (+ 5.21% points/year; 95% CI: 2.08–8.03; FDR-*p* = 0.002). Ceftriaxone and piperacillin-tazobactam resistance increased significantly in *E. coli*, while carbapenem resistance increased significantly in both species. Fluoroquinolone resistance also showed significant upward trends in *E. coli* and *K. pneumoniae*. Among aminoglycosides, amikacin resistance increased significantly in both species, whereas gentamicin resistance increased significantly only in *E. coli*. No significant temporal trend was observed for trimethoprim-sulfamethoxazole resistance in either species.

**Conclusion:**

UTIs remain common, with *E. coli* and *K. pneumoniae* showing increasing resistance trends, particularly against β-lactams, fluoroquinolones, carbapenems and aminoglycosides. These findings underscore the importance of ongoing surveillance and support the implementation of antimicrobial stewardship strategies.

## Introduction

Urinary tract infections (UTIs) remain a leading cause of bacterial illness worldwide, encompassing both community-acquired and healthcare-associated cases [1]. According to recent global estimates, the burden of UTIs has risen markedly over the past three decades. In 2019, the global incidence reached approximately 0.4 billion cases, representing a 60.4% increase compared to 1990 [2]. Over the same period, the number of deaths attributed to UTIs increased from nearly 98,590 in 1990 to 236,790 in 2019, corresponding to an approximate 140% rise, underscoring the growing clinical and public health significance of these infections [3]. This growing epidemiological burden has been further exacerbated by the escalating threat of antimicrobial resistance (AMR), which has emerged as one of the most pressing global health challenges of the 21st century [4]. In recognition of its potential catastrophic impact, the *Review on Antimicrobial Resistance* projected that, if current trends persist, AMR could become responsible for approximately 10 million deaths annually by 2050 surpassing deaths due to cancer and placing an immense financial and social strain on global health systems [5]. In this context, UTIs represent a relevant clinical setting for examining AMR, as they are frequently caused by highly adaptable bacterial species such as *Escherichia coli* and *Klebsiella pneumoniae*.

Understanding the molecular mechanisms that sustain their resistance and clinical persistence is therefore essential, recent studies have shown that resistance in *E. coli* is largely driven by the acquisition of plasmid and transposon-mediated resistance genes, including those encoding extended-spectrum β-lactamases and carbapenemase, together with efflux pump overexpression, porin alterations, target-site mutations, biofilm formation, and stress-response pathways [6]. In *k. pneumonia*, resistance is supported by a combination of intrinsic and acquired mechanisms, including chromosomal β-lactamases, porin loss, efflux pump activity, biofilm formation [7, 8], and the acquisition of transferable β-lactamase genes such as *bla*SHV, *bla*TEM, *bla*CTX-M, *bla*KPC, *bla*NDM, *bla*VIM, and *bla*OXA [9–13]. Fluoroquinolone resistance is mainly associated with mutations in DNA gyrase and topoisomerase IV [14]. While aminoglycoside resistance may involve aminoglycoside-modifying enzymes and 16 S rRNA methylases [15]. Fosfomycin resistance may also be influenced by *fosA*-related mechanisms [14]. Collectively, these mechanisms highlight the genomic plasticity of these uropathogens and their ability to persist under antimicrobial pressure.

Beyond these mechanistic insights, monitoring temporal resistance trends is essential to understand AMR evolution and its clinical impact over time. Over the past decade, longitudinal analyses have consistently demonstrated an upward trajectory in AMR among uropathogenic *E. coli* and *K. pneumoniae* worldwide. Yet, despite this growing global concern, the Middle East and North Africa (MENA) region remains critically underrepresented in resistance surveillance efforts, with recent reports emphasizing a pronounced paucity of epidemiological data [16].

In Morocco, evidence concerning the AMR profiles of uropathogenic *Enterobacteriaceae* is particularly scarce, reflecting significant gaps in systematic monitoring and microbiological investigation [17, 18]. Alarmingly, emerging data point to an increasing prevalence of β-lactamase and carbapenemase-producing isolates, mirroring the global expansion of multidrug-resistant phenotypes [19, 20]. Against this background, the present study aims to determine the prevalence, the epidemiological and microbiological characteristics of UTIs, and to assess AMR trends at the University Hospital Center Mohammed VI of Marrakech over a five-year period.

## Materials and methods

### Study design and period

Retrospective cross-sectional study, January 2020 - December 2024.

### Setting

University Hospital Center (CHU) Mohammed VI, a 1,548-bed tertiary hospital in Marrakech.

### Population

All patients with culture-confirmed UTIs (*n* = 8,231). Exclusion criteria included missing demographic or specimen date. To minimize potential bias due to repeated sampling, duplicate isolates were excluded, and only the first positive isolate per patient per infectious episode was retained for analysis.

### Microbiological methods

Urine samples were processed using standard microbiological techniques. Bacterial identification was performed by matrix-assisted laser desorption ionization time of flight mass spectrometry (MALDI-TOF MS, Bruker^®^). AST was carried out using the disk diffusion method and the BD Phoenix^®^ automated system. For consistency across the study period, AST results were rechecked and interpreted using a single harmonized CA-SFM/EUCAST 2025 guideline version, applied consistently to all study years. Extended-spectrum β-lactamase (ESBL) production was assessed phenotypically using the double-disk synergy test or the BD Phoenix^®^ automated ESBL detection system, according to CA-SFM/EUCAST recommendations.

### Data analysis

All statistical analyses were conducted using R software (version 4.5.1). The dataset included demographic variables, clinical department of origin, bacterial species, and antimicrobial susceptibility testing results. Counts and proportions were used to summarize categorical variables. Annual resistance rates were calculated for each bacterial species-antibiotic combination as the proportion of resistant isolates among the total number of tested isolates.

Temporal trends in annual resistance rates were assessed using beta-regression models with a logit link function, with calendar year included as a continuous predictor. Beta regression was selected because resistance rates are proportions bounded between 0 and 1 [21]. Trend estimates were expressed as annual percentage-point changes (PP/year), with corresponding 95% confidence intervals (CIs) derived from the fitted models. To account for multiple comparisons, *p*-values were adjusted using the Benjamini-Hochberg false discovery rate (FDR) procedure, and FDR-adjusted *p*-values were reported. Statistical significance was defined as an FDR-adjusted *p*-value < 0.05 [22].

## Results

### Epidemiological and microbiological profile of urinary tract infections

Over a five-year period, the microbiology department processed a total of (62,788) urine specimens. Of these, (8,231) yielded positive cultures, corresponding to a prevalence rate of 13.10%. This figure reflects the overall burden of UTIs within the studied population and serves as a key indicator of infection frequency over time. The prevalence of positive cultures was higher among female patients 59.6% compared to males 41.4% (Table 1). In terms of age distribution, adults aged over 60 years constituted the largest demographic group 30.3%, followed by children under 15 years 26.9% and individuals aged 40–59 years 21.7%. Patients aged 20–39 years accounted for 17.7%, whereas adolescents aged 15–19 years represented the smallest proportion 3.3%. Regarding sex distribution, women predominated across all age groups below 59 years. However, in the group aged over 60 years, the proportion of men 51.3% was slightly higher than that of women 48.7% (Table 1).

**Table 1:** Demographic characteristics of patients with positive urine cultures and distribution of bacterial isolates by hospital service

Section	Variable / Category	Subcategory	Frequency and (%)
A. Demographic characteristics			
	Total samples		62,788
	Total positive samples		8,231 (13.1%)
	Women		4,908 (59.62%)
	Men		3,323 (40.37%)
	Age distribution by sex		
		< 15 years (Men)	981 (44.2%)
		< 15 years (Women)	1237 (55.8%)
		15–19 years (Men)	85 (30.9%)
		15–19 years (Women)	190 (69.1%)
		20–39 years (Men)	350 (24%)
		20–39 years (Women)	1108 (76%)
		40–59 years (Men)	627 (35.1%)
		40–59 years (Women)	1160 (64.9%)
		≥ 60 years (Men)	1280 (51.3%)
		≥ 60 years (Women)	1213 (48.7%)
	Prevalence by service		
		Emergency	3695 (44.9%)
		Medicine	2328 (28.3%)
		Surgery	826 (10%)
		Intensive care	717 (8.7%)
		Pediatrics	665 (8.1%)
B. Bacterial isolates by service			
	Emergency	*E. coli*	2136 (79.11%)
		*K. pneumoniae*	564 (20.89%)
	Intensive Care	*E. coli*	160 (82.05%)
		*K. pneumoniae*	35 (17.95%)
	Medicine	*E. coli*	1177 (87.97%)
		*K. pneumoniae*	161 (12.03%)
	Pediatrics	*E. coli*	166 (64.34%)
		*K. pneumoniae*	92 (35.66%)
	Surgery	*E. coli*	303 (82.11%)
		*K. pneumoniae*	66 (17.89%)

The majority of isolates were recovered from specimens originating in the emergency department 44.9%, followed by internal medicine 28.3%, surgical wards 10%, pediatrics 8.7%, and intensive care units 8.1% (Table 1).

The distribution of *E. coli* and *K. pneumoniae* across hospital departments revealed notable variations. The highest proportion of *E. coli* isolates was observed in the Medicine department, where it accounted for 88% of cases. Comparable percentages were recorded in the Intensive Care Unit and the Surgery department, both reaching 82.1%. In contrast, the Pediatrics department showed the highest relative proportion of *K. pneumoniae*, which represented 35.7% of the isolates in this service. These findings indicate that while *E. coli* predominates in most clinical departments, *K. pneumoniae* exhibits a greater relative burden in pediatric patients (Table 1).

**Fig. 1:**
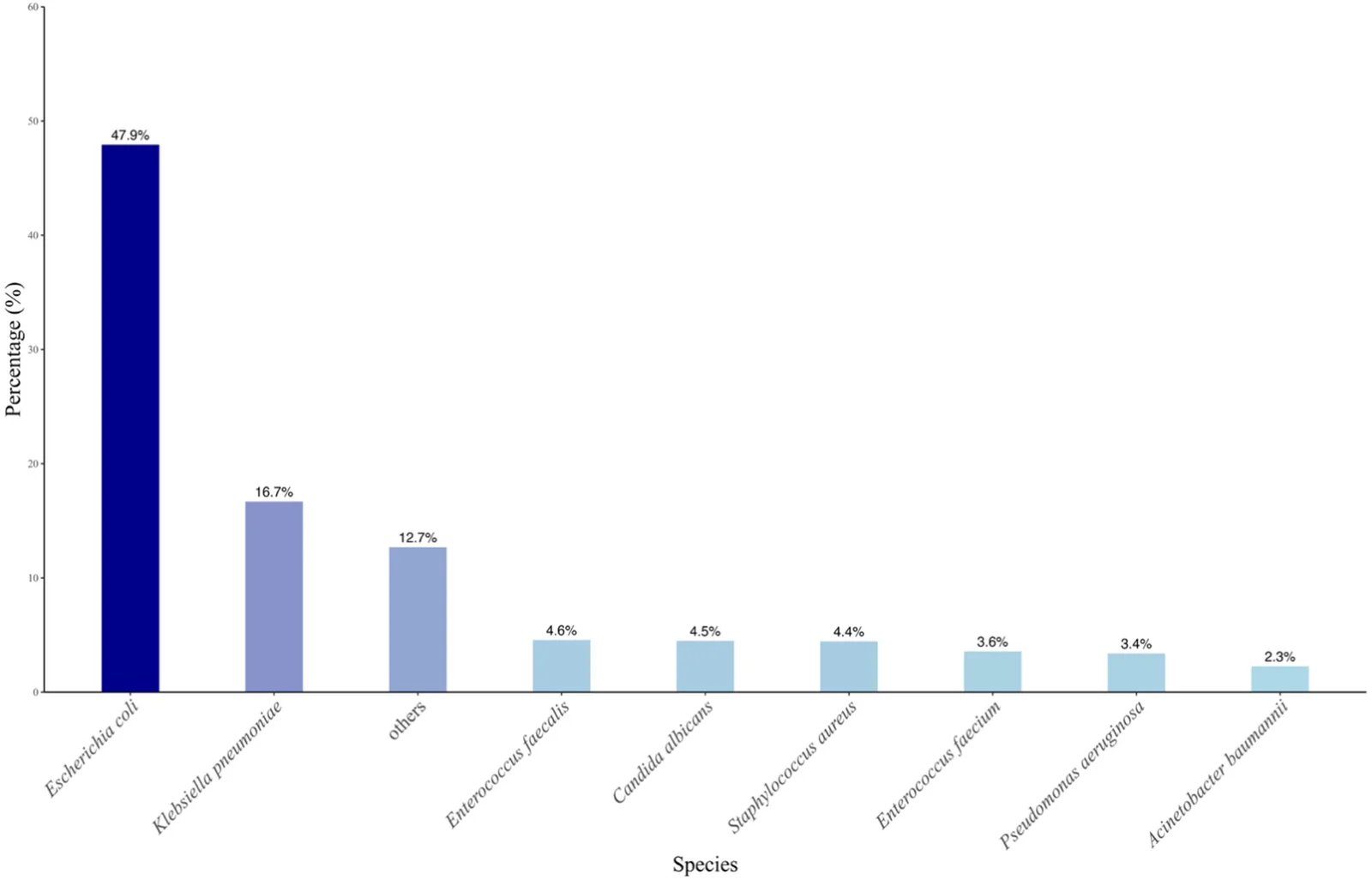
Prevalence of the most common pathogens causing urinary tract infection. Microorganisms with an individual prevalence below 2% were grouped under the category “Others”

Overall, among the 8,231 positive cultures analyzed, 82 species were identified. The most frequently isolated pathogens were *E. coli* 47.9%, *K. pneumoniae* 16.7%, and *Enterococcus spp.* 4.6%. Microorganisms with an individual prevalence below 2% were grouped under the category “Others,” comprising 12.7% of all isolates, while *Candida albicans* accounted for 4.5%. (Fig. 1).

This distribution highlights the marked predominance of Enterobacteriaceae, particularly *E. coli* and *K. pneumoniae*, as the principal etiological agents of UTIs in our cohort.

### Annual resistance rates and temporal trends in antimicrobial resistance among *Escherichia coli* and *Klebsiella pneumoniae* urinary isolates

Annual resistance rates for the main antimicrobial agents tested against *E. coli* and *K. pneumoniae* from 2020 to 2024 are presented in Table 2.

**Table 2:** Annual antimicrobial resistance rates of *E.coli (EC)* and *K.pneumoniae (KP)* urinary isolates to selected antibiotics, 2020–2024. Amoxicillin resistance was assessed only in *E. coli*, as *K. pneumoniae* is intrinsically resistant to aminopenicillins

Antibiotic class	Antibiotic	Isolate	2020 *EC* (*n* = 600) *KP *(*n* =166)	2021 *EC* (*n* = 846) *KP* (*n* = 302)	2022 *EC* (*n* = 1057) *KP* (*n* = 370)	2023 *EC* (*n* = 789) *KP* (*n* = 318)	2024 *EC* (*n* = 713) *KP* (*n* = 244)
β-lactams	Amoxicillin	*E. coli*	66%	67%	66%	67%	67%
	Amoxicillin-clavulanate	*E. coli*	27%	28%	41%	53.3%	45%
		*K. pneumoniae*	51%	41%	58%	62%	67%
	Ceftriaxone	*E. coli*	11%	10%	11%	14%	17%
		*K. pneumoniae*	44%	34%	36%	43.5%	58%
	Piperacillin-tazobactam	*E. coli*	10%	4%	6%	13%	15%
		*K. pneumoniae*	46%	27%	30%	45%	52%
	Ertapenem	*E. coli*	2%	2%	3%	5%	7%
		*K. pneumoniae*	17%	14%	17%	23%	28%
	Imipenem	*E. coli*	0.5%	0.6%	0.8%	1%	8%
		*K. pneumoniae*	10%	8%	10%	12%	16%
Fluoroquinolones	Fluoroquinolones	*E. coli*	28%	18%	32%	36%	38%
		*K. pneumoniae*	45%	37%	44%	52%	58%
Aminoglycosides	Gentamicin	*E. coli*	7%	3.5%	6%	9%	15%
		*K. pneumoniae*	37%	23%	20%	30%	46%
	Amikacin	*E. coli*	0.6%	0%	0.2%	0.8%	4%
		*K. pneumoniae*	0%	1%	3%	5%	15%
Folate pathway inhibitors	Trimethoprim-sulfamethoxazole	*E. coli*	37%	18%	32.3%	35%	40%
		*K. pneumoniae*	46%	40%	34%	42%	53%

Amoxicillin resistance in *E. coli* remained consistently high and stable over the study period, ranging from 66% to 67%, this antibiotic was not assessed for *K. pneumoniae* because of intrinsic resistance.

Resistance to amoxicillin-clavulanate increased in both species, from 27% to 45% in *E. coli* and from 51% to 67% in *K. pneumoniae*. Ceftriaxone resistance showed a gradual increase in *E. coli* from 11% to 17%, whereas in *K. pneumoniae* it fluctuated initially before reaching 58% in 2024. Piperacillin-tazobactam resistance remained lower in *E. coli*, increasing from 10% to 15%, while *K. pneumoniae* showed marked fluctuations, with resistance reaching 52% in 2024.

Carbapenem resistance increased over time in both species. Ertapenem resistance rose from 2% to 7% in *E. coli* and from 17% to 28% in *K. pneumoniae*, while imipenem resistance increased from 0.5% to 8% and from 10% to 16%, respectively. Fluoroquinolone resistance also showed an overall upward pattern, reaching 38% in *E. coli* and 58% in *K. pneumoniae* by 2024. Among aminoglycosides, gentamicin resistance increased from 7% to 15% in *E. coli* and from 37% to 46% in *K. pneumoniae*. Amikacin resistance remained low in *E. coli* but increased to 4% in 2024, while a more pronounced rise was observed in *K. pneumoniae*, from 0% to 15%. Finally, trimethoprim-sulfamethoxazole resistance fluctuated during the study period but remained high in 2024, reaching 40% in *E. coli* and 53% in *K. pneumoniae*.

**Fig. 2:**
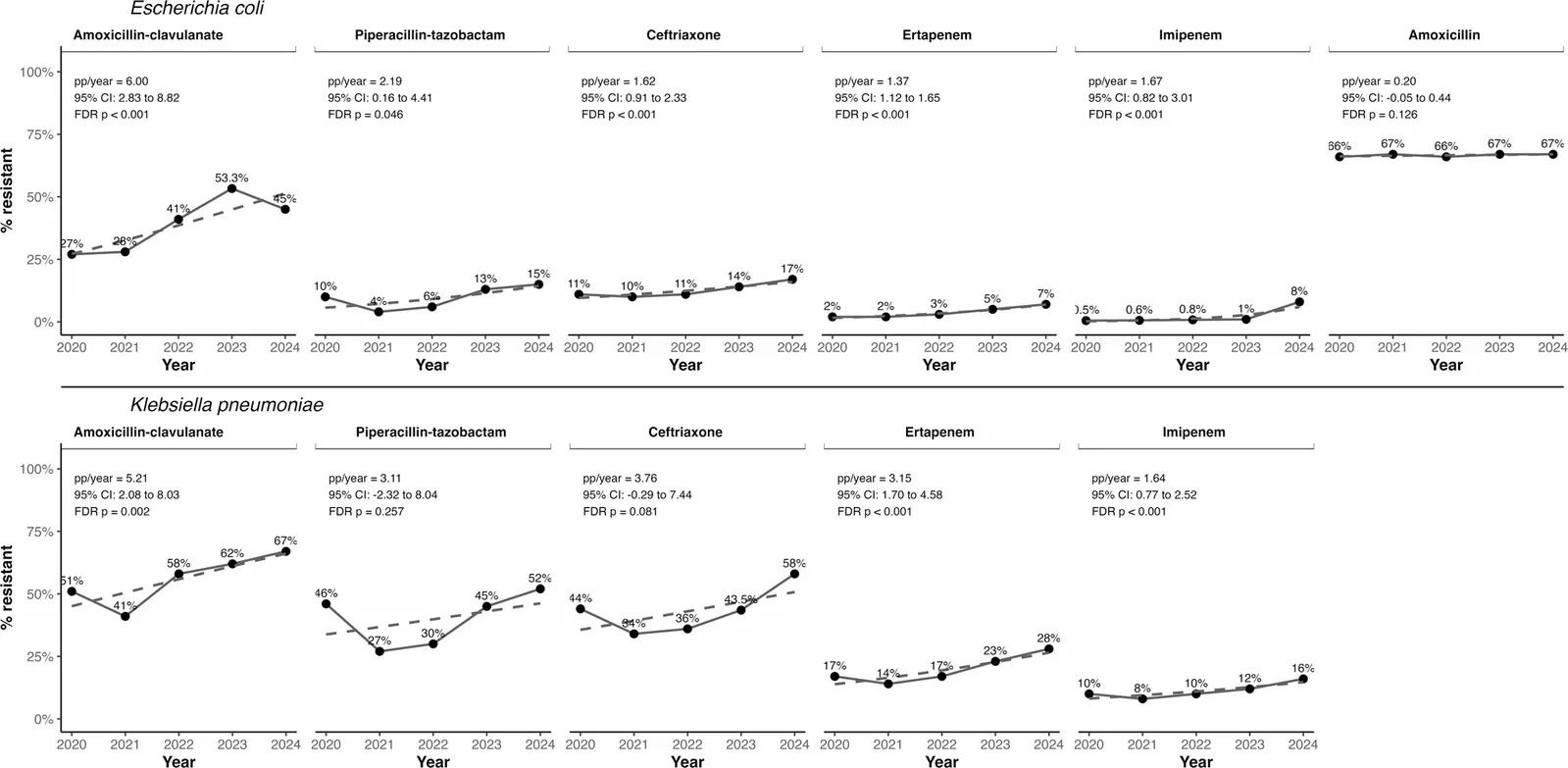
Temporal trends in β-lactam resistance among *Escherichia coli* and *Klebsiella pneumoniae* urinary isolates, 2020–2024. Annual resistance rates are shown as observed percentages. Dashed lines represent beta-regression fitted trends. Trends are expressed as percentage-point change per year (pp/year) with 95% confidence intervals and Benjamini-Hochberg false discovery rate-adjusted p-values (FDR p-values). As *K. pneumoniae* is intrinsically resistant to amoxicillin, this antibiotic was not included in the trend analysis for this species

**Fig. 3:**
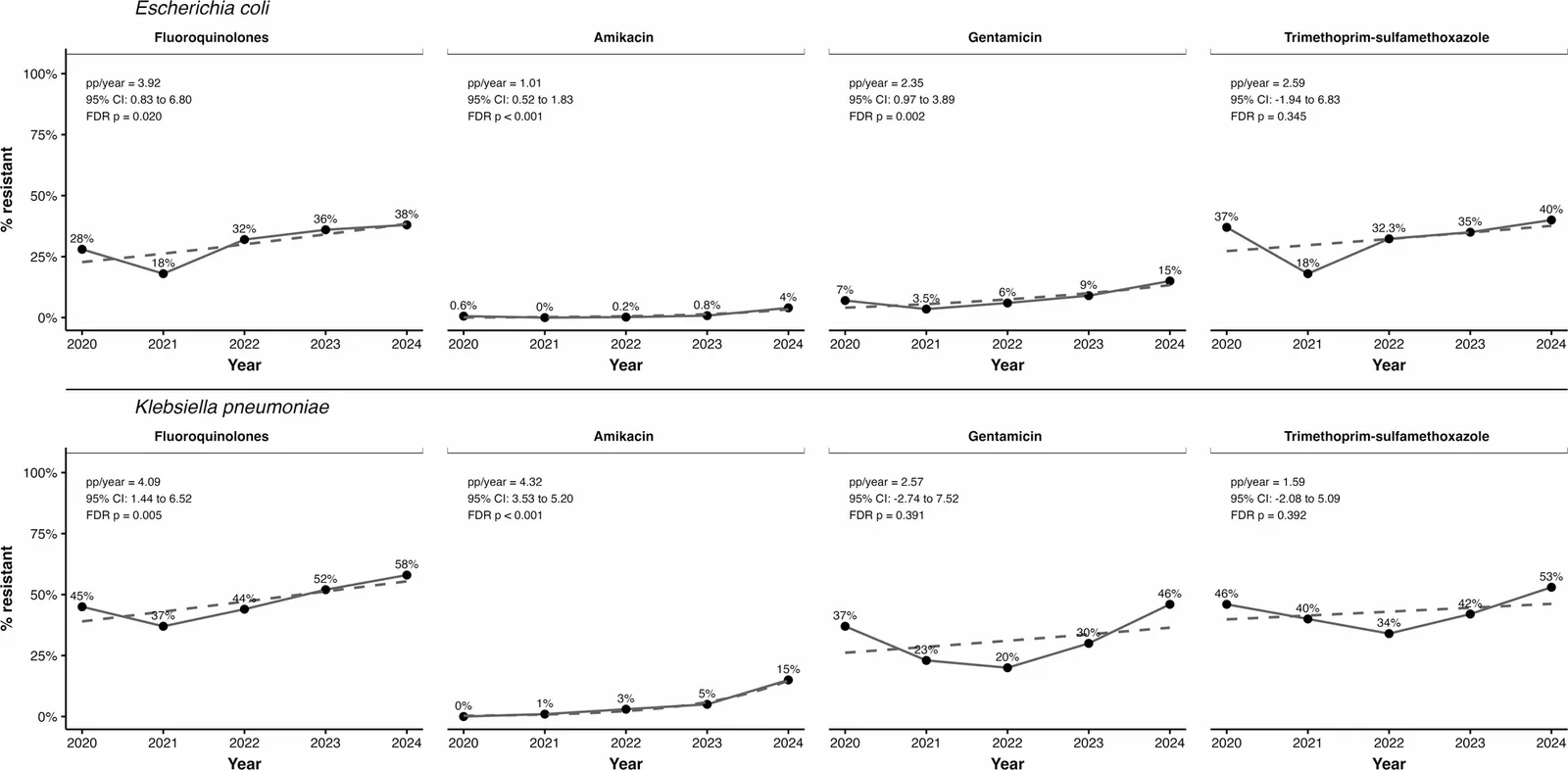
Temporal trends in non-β-lactam resistance among *Escherichia coli* and *Klebsiella pneumoniae* urinary isolates, 2020–2024. Annual resistance rates are shown as observed percentages. Dashed lines represent beta-regression fitted trends. Trends are expressed as percentage-point change per year (pp/year) with 95% confidence intervals and Benjamini-Hochberg false discovery rate-adjusted *p*-values (FDR *p*-values)

Temporal trends in antimicrobial resistance are presented separately for β-lactam agents in Fig. 2 and non-β-lactam agents in Fig. 3.

Amoxicillin resistance in *E. coli* remained consistently high over the study period, with no significant temporal change (+ 0.20% points per year; 95% CI: -0.05 to 0.44; FDR-*p* = 0.126), while this analysis was not performed for *K. pneumoniae* due to its intrinsic resistance to amoxicillin.

For amoxicillin-clavulanate, resistance increased significantly in both *K. pneumoniae* and *E. coli*, with estimated annual increases of + 5.21% points per year (95% CI: 2.08–8.03; FDR-*p* = 0.002) and + 6.00% points per year (95% CI: 2.83–8.82; FDR-*p* < 0.001), respectively.

Regarding third-generation cephalosporins, ceftriaxone resistance increased significantly in *E. coli* (+ 1.62% points per year; 95% CI: 0.91–2.33; FDR-*p* < 0.001), whereas the increase observed in *K. pneumoniae* did not reach statistical significance (+ 3.76% points per year; 95% CI: -0.29 to 7.44; FDR-*p* = 0.081). Similarly, piperacillin-tazobactam resistance increased significantly in *E. coli* (+ 2.19% points per year; 95% CI: 0.16–4.41; FDR-*p* = 0.046), but not in *K. pneumoniae* (+ 3.11% points per year; 95% CI: -2.32 to 8.04; FDR-*p* = 0.257). For carbapenems, both ertapenem and imipenem showed significant increases in phenotypic resistance in the two species. In *K. pneumoniae*, resistance increased by + 3.15% points per year for ertapenem (95% CI: 1.70–4.58; FDR-*p* < 0.001) and by + 1.64% points per year for imipenem (95% CI: 0.77–2.52; FDR-*p* < 0.001). In *E. coli*, the corresponding increases were + 1.37% points per year for ertapenem (95% CI: 1.12–1.65; FDR-*p* < 0.001) and + 1.67% points per year for imipenem (95% CI: 0.82–3.01; FDR-*p* < 0.001). Figure 2.

A significant upward trend was also observed for fluoroquinolone resistance in both species, with increases of + 4.09% points per year in *K. pneumoniae* (95% CI: 1.44–6.52; FDR-*p* = 0.005) and + 3.92% points per year in *E. coli* (95% CI: 0.83–6.80; FDR-*p* = 0.020). Among aminoglycosides, amikacin resistance increased significantly in both *K. pneumoniae* (+ 4.32% points per year; 95% CI: 3.53–5.20; FDR-*p* < 0.001) and *E. coli* (+ 1.01% points per year; 95% CI: 0.52–1.83; FDR-*p* < 0.001). Gentamicin resistance increased significantly in *E. coli* (+ 2.35% points per year; 95% CI: 0.97–3.89; FDR-*p* < 0.002), whereas no significant trend was observed in *K. pneumoniae* (+ 2.57% points per year; 95% CI: -2.74 to 7.52; FDR-*p* = 0.391). Trimethoprim-sulfamethoxazole resistance showed no statistically significant temporal trend in either *K. pneumoniae* (+ 1.59% points per year; 95% CI: -2.08 to 5.09; FDR-*p* = 0.392) or *E. coli* (+ 2.59% points per year; 95% CI: -1.94 to 6.83; FDR-*p* = 0.345). Figure 3.

## Discussion

This study examined the prevalence and AMR patterns of UTIs in a university hospital in Marrakech between 2020 and 2024. The prevalence of positive urine cultures was 13.1%, closely aligning with the 11% reported in a large Moroccan cohort of 37,394 urine samples collected between 2018 and 2020 [23], and comparable to the 18% observed by [24]. Higher-positivity rates have been described in Uganda 32.3% [25], Italy 31% [26], and Saudi Arabia 23.4% [27]. Lower rates, below 9.8%, were reported in India and Bangladesh (Mohapatra et al., [28]; Rahman et al., [29]). This variability is unlikely to reflect a single determinant. Differences in geographical and epidemiological contexts, methodological approaches, and sociocultural determinants, including hygiene practices, health literacy, and sex distribution can all shift the measured prevalence of UTIs [30].

Women were disproportionately affected in our cohort (59.6% vs. 41.4%; sex ratio 0.69), consistent with national data from Morocco (sex ratio 0.46) [24] and with international reports showing a female predominance (65.4% and 80% in Iran and Italy respectively) [31, 32]. A smaller gap was noted in Saudi Arabia (52.1% vs. 47.9%) [27]. Multiple studies have delineated a range of anatomical, physiological, and behavioral determinants that predispose certain populations to UTIs. In women, these include the close anatomical proximity of the urethra to the anus, the comparatively less acidic vaginal milieu, and the shorter and wider urethral canal, all of which facilitate ascending bacterial colonization. Furthermore, sexual activity, urinary incontinence, and inadequate hygienic practices have consistently been reported as additional risk factors contributing to the elevated burden of infection [33–35].

Most positive cultures originated from the emergency department 44.9%, followed by internal medicine 28.3%, surgery 10%, pediatrics 8.7%, and intensive care units 8.1%. This distribution parallels previous Moroccan reports, where emergency and medical wards were the leading contributors to UTI diagnoses [23, 36]. The predominance of positive cultures in the emergency department underscores its pivotal role in the initial assessment of patients presenting with acute urinary symptoms and in the early detection of community-acquired UTIs.

The bacterial distribution confirmed the central role of Enterobacteriaceae particularly *E. coli* and *K. pneumoniae* in UTIs. *E. coli* predominated in internal medicine 88%, exceeding the 66.5% reported by [23]. Elevated proportions were also observed in the ICU 82.05%, surgery 82.1%, and emergency 79.1%, surpassing the 52–74% range reported in the same settings. *K. pneumoniae* accounted for 17.95% of isolates in ICU and 17.89% for surgical wards, consistent with the findings of [37] (20.8% and 16.3%, respectively). Notably, *K. pneumoniae* exhibited the highest relative proportion in pediatrics 35.7%, although [23] reported even greater proportions in pediatrics 45% and neonatology 52%. These results therefore suggest two complementary patterns, broad dominance of *E. coli* across departments, and a stronger relative contribution of *K. pneumoniae* in younger hospital populations.

In this context, the predominance of *E. coli* and *K. pneumoniae* across hospital departments should be interpreted alongside their antimicrobial resistance profiles, as these patterns directly influence empirical treatment choices and local surveillance priorities. Accordingly, the present findings show substantial variation in antimicrobial resistance according to both bacterial species and antibiotic class.

Among β-lactams, amoxicillin resistance in *E. coli* remained consistently high throughout the study period, in line with previous Moroccan studies [24], although lower than the median of 22 studies in a national literature synthesis [38]. This antibiotic was not evaluated for *K. pneumoniae*, given its intrinsic resistance to aminopenicillins.

Resistance to amoxicillin-clavulanate increased in both species. In *E. coli*, resistance ranged from 27% to 45%, remaining lower than the rate reported in the Moroccan literature synthesis [39] 64% and in Bangladesh [29] 91.8%. These levels were also close to those reported by [40], who observed amoxicillin-clavulanate resistance rates ranging from 45% to 54% in *E. coli*. Importantly, beta-regression analysis confirmed a significant upward trend, with an estimated annual increase of + 6.00% points per year (FDR-*p* < 0.001), indicating a marked reduction in susceptibility over the study period. In *K. pneumoniae*, resistance to amoxicillin-clavulanate ranged from 51% to 67%, closely aligning with rates reported in Morocco [24] 53.8% and Saudi Arabia [27] 50.0%. However, the range observed in our study was lower than that reported by [40], who found resistance rates ranging from 57% to 89%. Beta-regression analysis also showed a significant increase resistance, with an estimated annual rise of + 5.21% points per year (FDR-*p* = 0.002). Taken together, these findings indicate that amoxicillin-clavulanate resistance is increasing significantly in both species, which may limit the reliability of this agent for empirical treatment.

Resistance to third-generation cephalosporins, represented here by ceftriaxone, differed markedly between the two species. In *E. coli*, ceftriaxone resistance remained moderate, ranging from 10% to 17%, and was lower than the Moroccan literature median of 28.7% [38], the rate reported from Bangladesh 65.2% [29], the rate reported in Oman 46% [41], and the cefotaxime resistance rates reported in a Bulgarian study 36%-38% [42]. Despite these comparatively lower levels, beta-regression analysis showed a significant increasing trend, with an estimated annual rise of + 1.62% points per year (FDR-*p* < 0.001). This increase was greater than that reported by [42] for cefotaxime resistance in *E. coli* (+ 0.823% points per year). In contrast, *K. pneumoniae* showed substantially higher ceftriaxone resistance levels 44% to 58%, which were comparable to Moroccan estimates 58.6% [38], slightly higher than the prevalence reported from Bangladesh 40.71% [29] and Oman 39% [41], but lower than the cefotaxime resistance rates 65–67% [42], beta-regression estimated a positive annual change ceftriaxone resistance (+ 3.76% points per year), but this trend did not remain statistically significant (FDR-*p* = 0.081). The higher resistance burden observed in *K. pneumoniae* is compatible with the circulation of extended-spectrum β-lactamase-producing strains, however, ceftriaxone resistance should not be interpreted as a direct proxy for ESBL production, as other resistance mechanisms may also contribute.

Piperacillin-tazobactam resistance remained relatively moderate in *E. coli*, ranging from 10% to 15%, although beta-regression analysis indicated a statistically significant upward trend over time (+ 2.19% points per year; FDR-*p* = 0.046). These levels were higher than the 5% resistance reported in Oman [41], but remained substantially lower than the 40% resistance in Moroccan studies [43], which was based exclusively on ESBL-producing *E. coli* isolates. In *K. pneumoniae*, piperacillin-tazobactam resistance was markedly higher, ranging from 46% to 52%. Although beta-regression estimated a positive annual change (+ 3.11% points per year), this trend was not statistically significant (FDR-*p* = 0.257). The resistance levels observed were higher than those reported in Oman 28% [41] and in India 26% [44]. These results suggest that piperacillin-tazobactam retains relatively better activity against *E. coli* than against *K. pneumoniae*.

Carbapenems remained among the most active β-lactam agents, especially against *E. coli*, although significant increasing trends in phenotypic resistance were observed for both ertapenem and imipenem in the two species.

For ertapenem, phenotypic resistance remained relatively low in *E. coli*, increasing from 2% to 7%. These levels were close to the low carbapenem resistance reported in the Moroccan literature synthesis, where the median resistance across 21 studies was 3.0% [38], and were also comparable to the 2% ertapenem resistance reported in Oman [41]. However, beta-regression analysis showed a significant upward trend, with an estimated annual increase of + 1.37% points per year (FDR-*p* < 0.001). In *K. pneumoniae*, ertapenem resistance was substantially higher, ranging from 17% to 28%, and was close to the 19% reported in Oman [41]. Beta-regression also confirmed a significant increase in ertapenem resistance (+ 3.15% points per year; FDR-*p* = 0.001). These findings indicate that ertapenem phenotypic resistance remains more frequent in *K. pneumoniae* than in *E. coli*, with a significant upward trend in both species.

For imipenem, phenotypic resistance also remained comparatively low in *E. coli*, ranging from 1% to 8%, and was generally close to the low resistance levels reported in Moroccan studies [38], although slightly higher than the 2% imipenem resistance reported by [41]. Beta-regression analysis revealed a significant annual increase in imipenem resistance (+ 1.67% points per year; FDR-*p* < 0.001). In *K. pneumoniae*, imipenem resistance was higher, ranging from 10% to 16%, and remained close to the 17% reported by [41]. A significant increasing trend was also observed (+ 1.64% points per year; FDR-*p* = 0.001). Although the resistance levels observed in our study were lower than the very high carbapenem resistance rates reported in some settings, including Vietnam 62.2% [45] and Saudi Arabia 44.7% [27]. The significant upward trends in phenotypic resistance observed for both carbapenems warrant clinical attention. Since carbapenems are commonly reserved for severe infections caused by multidrug-resistant Enterobacterales, even moderate increases in resistance may progressively limit therapeutic options.

For non-β-lactam agents, fluoroquinolone resistance showed an overall increasing pattern in the both species. In *E. coli*, resistance reached 38%, exceeding rates reported from El Jadida 20% [46] and Rabat 27% [47], remaining close to the Moroccan literature median 34% [38], and being higher than values reported in the United States 10% [48] but lower than those observed in China [49]. This increase was supported by beta-regression analysis, which showed a significant annual rise of + 3.92% points per year (FDR-*p* = 0.020). interestingly, this trend contrasts with the finding by [42], who reported a significant decreasing trend in fluoroquinolone resistance, from 42% to 39% between 2017 and 2022, with an estimated annual change of -0.542% points per year. In *K. pneumoniae*, fluoroquinolone resistance ranged from 45 to 58%, comparable to Moroccan estimates [24, 38], although lower than the high prevalence reported in Vietnam 80.7% [45]. Beta-regression confirmed a significant increasing trend resistance, with an annual increase of + 4.09% points per year (FDR-*p* = 0.005). This upward pattern is consistent with [42], who also reported a significant increase in fluoroquinolone resistance, from 59% to 74%, corresponding to + 2.93% points per year. The high baseline resistance and significant upward trends suggest that fluoroquinolones have limited reliability as empirical options.

Aminoglycosides showed a more favorable resistance profile than the other non-β-lactam agents. For amikacin, resistance remained low in *E. coli*, increasing from 1% to 4%, and was comparable to the low rate 2% reported by [41]. In *K. pneumoniae*, amikacin also retained comparatively good activity, although resistance increased over time and reached 15% in 2024. This rate remained lower than the 20% reported by [41]. However, beta-regression analysis showed significant upward trends for amikacin resistance in both species, with a moderate annual increase in *E. coli* (+ 1.01% points per year; significant FDR-p) and a more marked increase in *K. pneumoniae* (+ 4.32% points per year; significant FDR-p). For gentamicin, resistance was higher than that observed for amikacin in both species. *In E. coli*, gentamicin resistance ranged from 7% to 15%, which is close to the 13% reported by [41]. This relatively low level is consistent with previous reports showing preserved aminoglycoside activity [50]. Nevertheless, beta-regression analysis revealed a significant increase in gentamicin resistance (+ 2.35% points per year; FDR-*p* = 0.002), suggesting a gradual reduction in susceptibility over time. *In K. pneumoniae*, gentamicin resistance was higher and more variable than in *E. coli*, and was also higher than the 20% reported by [41]. However, the estimated annual increase in *K. pneumoniae* was not statistically significant (+ 2.57% points per year; FDR-*p* = 0.391). These results indicate that amikacin remains more active than gentamicin against both species, although the significant upward trends observed for amikacin, especially in *K. pneumoniae*, highlight the need for continued monitoring.

Trimethoprim-sulfamethoxazole resistance remained substantial in both species, although the temporal trends were not statistically significant. In *E. coli*, resistance ranged from 32.3% to 40%, which was lower than the Moroccan literature median of 56% [38] and the rate reported from Bangladesh 56.9% [29], while being comparable to the 37% resistance reported by [41]. Although beta-regression estimated a positive annual change of + 2.59% points per year, this trend was not statistically significant (FDR-*p* = 0.345), suggesting that TMP-SMX resistance remained relatively stable over the study period despite persistently elevated levels. In *K. pneumoniae*, TMP-SMX resistance ranged from 46% to 52%, remaining higher than that observed in *E. coli*. These levels were close to the Moroccan estimate of 50.9% [38] and to data from Bangladesh 50.7% [29], but higher than the 35% resistance reported by [41]. Beta-regression analysis also showed a positive but non-significant annual change (+ 1.59% points per year; *p* = 0.392). Overall, these findings indicate that TMP-SMX resistance remains high in both species, but without a statistically supported increase over time in the present dataset.

On the whole, these findings highlight the predominance of *E. coli* and *K. pneumoniae* in UTIs, with increasing resistance to several key antibiotics. This pattern supports the need for continuous surveillance and regular updating of empirical treatment guidelines.

### Strengths and limitations of the study

This study has several strengths. First, it addresses an important gap in AMR surveillance data from North Africa by providing regional evidence on uropathogen distribution and antimicrobial resistance trends. Second, the large sample size, including 62,788 urine samples and 8,231 positive cultures, together with the five-year study period (2020–2024), allowed a robust assessment of UTI etiology and temporal AMR trends. Third, the study was conducted in a major tertiary-care hospital in the regio and relied on standardized microbiological procedures, including MALDI-TOF MS for bacterial identification. Moreover, AST results were harmonized using a single CA-SFM/EUCAST guideline version, ensuring consistent interpretation across the study period. Finally, the use of beta-regression models strengthened the methodological approach and supported a more rigorous interpretation of resistance trends over time.

Nevertheless, this study has several limitations. First, its monocentric design may limit the generalizability of the findings to other regions or healthcare settings.

Second, due to the retrospective laboratory-based design, detailed clinical information, including symptomatology, pregnancy status, and underlying comorbidities, was not available. The absence of these data prevented the identification of factors associated with the risk of UTIs and their potential influence on the observed antimicrobial resistance patterns.

Third, UTIs could not be reliably classified as community-acquired, hospital-acquired, or catheter-associated, which may have affected the epidemiological interpretation of resistance patterns.

Fourth, molecular characterization of resistant isolates was not performed; therefore, the genetic determinants underlying the observed resistance phenotypes, including carbapenem resistance, could not be identified.

Finally, the absence of data on prior antibiotic exposure, including self-medication practices, antibiotic class, dosage, and treatment duration, prevented assessment of the potential impact of antimicrobial use on uropathogen distribution and resistance profiles, and limited our ability to directly relate the observed resistance trends to antibiotic prescribing practices.

## Conclusion

This five-year retrospective study provides an updated overview of the epidemiological, microbiological, and antimicrobial resistance patterns of culture-confirmed UTIs at the University Hospital Center Mohammed VI of Marrakech. UTIs represented a sustained diagnostic burden, with a culture positivity rate of 13.10%, and were more frequently observed among women and older adults. *E. coli* and *K. pneumoniae* were the predominant uropathogens, confirming the major role of Enterobacteriaceae in the local epidemiology of UTIs. The temporal analysis revealed concerning increases in resistance among both species, particularly to amoxicillin-clavulanate, carbapenems, fluoroquinolones, and amikacin. In *E. coli*, significant upward trends were also observed for ceftriaxone, piperacillin-tazobactam, and gentamicin, whereas trimethoprim-sulfamethoxazole resistance remained high but did not show a statistically significant temporal increase. These findings emphasize the importance of continuous microbiological surveillance, regular updating of empirical treatment recommendations, and strengthened antimicrobial stewardship interventions to limit the progression of resistance in urinary pathogens.

## Data Availability

The datasets used and /or analyzed during the current study available from the corresponding author on reasonable request.

## Abbreviations

AMR: Antimicrobial resistance
AST: Antimicrobial susceptibility testing
CA-SFM: Comité de l’Antibiogramme de la Société Française de Microbiologie
CHU: Centre Hospitalier Universitaire
CI: Confidence interval
EUCAST: European Committee on Antimicrobial Susceptibility Testing
ESBL: Extended-spectrum β-lactamase
FDR-p: False discovery rate-adjusted *p*-value
MALDI-TOF MS: Matrix-assisted laser desorption/ionization time-of-flight mass spectrometry
MENA: Middle East and North Africa
pp/year: Percentage points per year
UTIs: Urinary tract infections
E. coli: Escherichia coli
K. pneumoniae: Klebsiella pneumoniae
β-lactams: Beta-lactams
DNA: Deoxyribonucleic acid
rRNA: Ribosomal ribonucleic acid
bla_SHV: β-lactamase gene encoding SHV-type enzymes
bla_TEM: β-lactamase gene encoding TEM-type enzymes
bla_CTX-M: β-lactamase gene encoding CTX-M-type enzymes
bla_KPC: β-lactamase gene encoding Klebsiella pneumoniae carbapenemase
bla_NDM: β-lactamase gene encoding New Delhi metallo-β-lactamase
bla_VIM: β-lactamase gene encoding Verona integron-encoded metallo-β-lactamase
bla_OXA: β-lactamase gene encoding OXA-type oxacillinase
aac(6′)-Ib-cr: Aminoglycoside acetyltransferase variant associated with fluoroquinolone resistance
armA: Aminoglycoside resistance methyltransferase gene
AcrAB-TolC: AcrAB-TolC multidrug efflux pump system
KpnEF: KpnEF multidrug efflux pump system
KpnO: Outer membrane protein associated with antimicrobial resistance in K. pneumoniae
fos: Fosfomycin resistance gene
